# Supplementary material for: South African University Staff and Students’ Perspectives, Preferences, and Drivers of Hesitancy Regarding COVID-19 Vaccines: A Multi-Methods Study
Source: Vaccines (Basel). 2022 Aug 4;10(8):1250. doi: 10.3390/vaccines10081250 (PMC9412872; doi:10.3390/vaccines10081250)
Supplement: Supplementary file 1 [file vaccines-10-01250-s001.zip › vaccines-1827235-supplementary.pdf]

DCE Choice Set 1 of 8

Which of the following models of COVID-19 vaccination do you most prefer?

Option A

Vaccination Location:
UKZN Campus

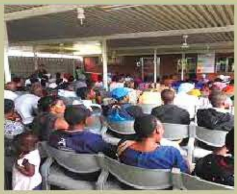

3 hours
waiting time at the vaccination site

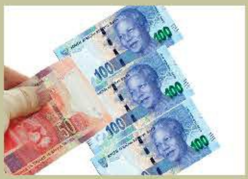

You get a R 350 incentive for vaccinating

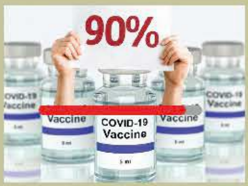

Vaccine is 90% (very) effective against severe illness and death

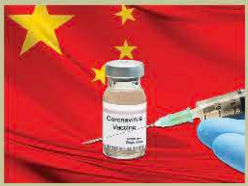

The vaccine comes from China

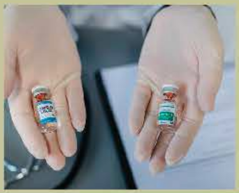

Two doses

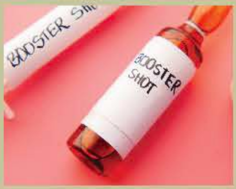

Booster vaccination required every 5 years

Option B

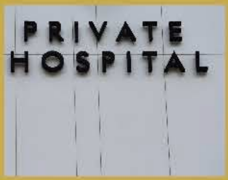

Vaccination Location:
Private Health Facility

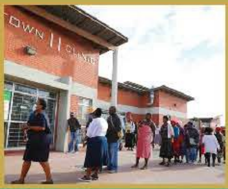

5 hours
waiting time at the vaccination site

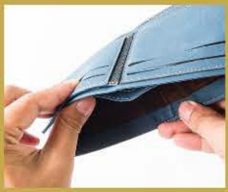

You get no incentive for vaccinating

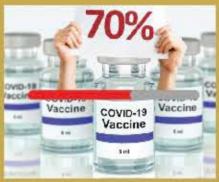

Vaccine is 70% (moderately) effective against severe illness and death

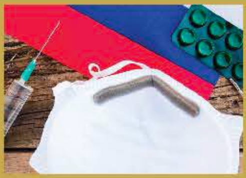

The vaccine comes from Russia

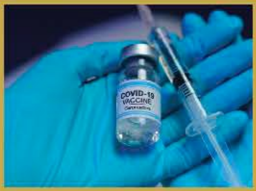

One dose

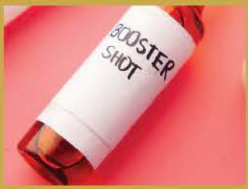

Annual booster vaccinations required
